# One‐Year Effectiveness of Upadacitinib in Perianal Crohn's Disease: A Real‐World GETAID Study

**DOI:** 10.1111/apt.70682

**Published:** 2026-04-27

**Authors:** Nicolas Richard, Philippe Seksik, Romain Altwegg, Maria Nachury, David Laharie, Stéphane Nancey, Benoît Coffin, Anne‐Laure Pelletier, Mathieu Uzzan, Aurélien Amiot, Morgane Amil, Lucine Vuitton, Mathurin Fumery, Anne Bozon

**Affiliations:** ^1^ Department of Gastroenterology University Rouen Normandie, INSERM, ADEN UMR1073, “Nutrition, Inflammation and Microbiota‐Gut‐Brain Axis”, CHU Rouen Rouen France; ^2^ Department of Gastroenterology CRSA, Sorbonne Université, INSERM UMRS‐938, Gut, Liver & Microbiome Research (GLIMMER) FHU, APHP, Saint‐Antoine Hospital Paris France; ^3^ Department of Gastroenterology CHU of Montpellier Montpellier France; ^4^ University of Lille, INSERM, Lille University Hospital, U1286 ‐ INFINITE ‐ Institute for Translational Research in Inflammation Lille France; ^5^ Department of Gastroenterology CHU de Bordeaux, Haut‐Lévêque Hospital, University of Bordeaux Bordeaux France; ^6^ Department of Gastroenterology Lyon‐Sud Hospital, CHU of Lyon Lyon France; ^7^ Clinique Ambroise Paré Hartman, Institut des MICI Neuilly sur Seine France; ^8^ Department of Gastroenterology CHU Bichat‐Claude Bernard Paris France; ^9^ Gastroenterology Department Henri Mondor Hospital, Fédération Hospitalo‐Universitaire TRUE InnovaTive theRapy for immUne disordErs, Paris Est Créteil University UPEC, Assistance Publique‐Hôpitaux de Paris (AP‐HP) Créteil France; ^10^ Department of Gastroenterology Hôpitaux Universitaires Bicêtre, AP‐HP, Université Paris Saclay Le Kremlin Bicêtre France; ^11^ Department of Gastroenterology La Roche Sur Yon Hospital La Roche Sur Yon France; ^12^ Department of Gastroenterology UMR Right Inserm 1098, University Marie et Louis Pasteur, Franche‐Comté Besançon France; ^13^ Department of Gastroenterology Amiens University Hospital And PeriTox, University of Picardie Amiens France

**Keywords:** Crohn's disease, fistulizing perianal Crohn's disease, upadacitinib

## Abstract

**Introduction:**

Upadacitinib (UPA) is effective for treating luminal Crohn's disease (CD), but data on perianal CD (pCD) remain limited.

**Methods:**

All consecutive patients with active pCD (primary and/or secondary lesions) treated with UPA across 13 French centres between September 2022 and August 2025 were included in a retrospective cohort study. Clinical remission was defined as absence of fistula drainage (spontaneous or on gentle pressure) and healing of anal ulcerations without initiation of new therapy. Clinical response was defined as ≥ 50% improvement in fistulas and/or ulcerations based on physician assessment. Clinical outcomes were analysed using non‐responder imputation, and magnetic resonance imaging (MRI) outcomes as observed.

**Results:**

Among the 59 patients included, 43 (73%) had fistulizing pCD and 16 (27%) isolated anal ulcerations. All patients were previously exposed to at least one biologic and 79% of those with fistulizing pCD underwent prior perianal surgery. In patients with fistulizing pCD, clinical remission was achieved in 11/43 (26%) and 11/43 (26%) patients at 6 and 12 months, respectively, clinical response in 21/43 (49%) and 13/43 (30%) patients. At 12 months, MRI response was documented in 9/13 (69%) and MRI remission in 1/13 (8%) patients with fistulizing pCD. Among patients with fistulizing pCD, no factors predicted clinical remission. Among patients with isolated anal ulcerations, complete healing occurred in 5/16 (31%) at 3 months, 4/16 (25%) at 6 months and 4/16 (25%) at 12 months.

**Conclusion:**

In this real‐world cohort of refractory pCD, UPA achieved clinical remission in one‐quarter of patients at 1 year.

## Graphical abstract

In a real‐world cohort of patients with highly refractory perianal Crohn's disease, upadacitinib induced clinical remission at 12 months in 26% of patients with fistulizing disease and 25% of those with isolated anal ulcerations.

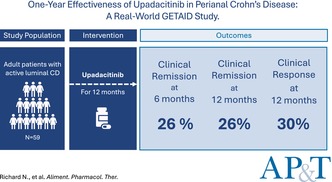

**Abbreviations**
95% CI: 95% confidence interval
AEs: adverse events
AO: as observed
BMI: body mass index
CD: Crohn's disease
HBI: Harvey Bradshaw index
IBD: inflammatory bowel disease
IQR: interquartile range
JAK: Janus kinases
MRI: magnetic resonance imaging
NRI: non‐responder imputation
pCD: perianal CD
PRO2: patient reported outcome 2
SES‐CD: simple endoscopic score in CD
TNF: tumour necrosis factor
US: ultrasound

## 1 Introduction

Crohn's disease (CD) is an inflammatory disease of the gastrointestinal tract, with perianal involvement occurring in approximately 20% of patients within 10 years of diagnosis [1]. Perianal CD (pCD) includes ulcerated primary lesions such as fissures and cavitating perianal ulcerations, and more frequently secondary lesions, corresponding to abscesses or fistulas [2, 3]. Life‐ and work‐related opportunities have been shown to be affected by pCD, with an intense and wide‐reaching impact, negatively affecting intimate, close, and social relationships [4].

Despite its burden and prevalence, evidence supporting medical management of pCD remains limited. In this setting, infliximab is the most effective therapy evaluated in dedicated randomized, placebo‐controlled trials, demonstrating efficacy for both induction and maintenance [5, 6]. However, clinical remission is achieved in only approximately one‐third of patients after 1 year [6], highlighting a critical unmet need for alternative therapeutic options following anti‐TNF failure.

Upadacitinib is an oral, reversible Janus kinase (JAK) inhibitor with preferential selectivity for JAK1, resulting in downregulation of multiple pro‐inflammatory cytokines implicated in CD pathogenesis [7, 8]. In phase 3, double‐blind, placebo‐controlled induction trials, upadacitinib has demonstrated superiority over placebo in achieving clinical remission and endoscopic response in patients with moderate‐to‐severe luminal CD [8]. In a subsequent *post hoc* analysis of patients including 56 patients with draining perianal fistula, 52 weeks of upadacitinib treatment was associated with higher rates of fistula drainage resolution compared with placebo (23%–29% depending on maintenance regimen versus 0%) [9].

Few studies have reported real‐world effectiveness data for upadacitinib in fistulizing pCD to date. A three‐patient case series reported promising results in severe post‐proctectomy pCD after approximately 2 years of follow‐up [10]. Our group previously published week 12 outcomes in a subgroup of 54 patients, showing clinical improvement in 67% and clinical remission in 14% of patients [11]. Consequently, additional real‐world effectiveness data are warranted.

The aim of this study was therefore to evaluate the 1‐year clinical effectiveness and safety of upadacitinib in patients with pCD and to identify potential predictors of therapeutic response.

## 2 Patients and Methods

### 2.1 Study Design and Patient Population

This multicentre retrospective cohort study was conducted across 13 French centres affiliated with the Groupe d'Étude Thérapeutique des Affections Inflammatoires du Tube Digestif (GETAID) between September 2022 and August 2025. Participating centres were asked to identify consecutive eligible patients through a review of their cohort databases. This study was conducted in accordance with the ethical principles of the Declaration of Helsinki and was registered in the French health data hub (No. 1670906), as required by national regulations. Data processing was declared to the Commission Nationale de I'Informatique et des Libertés (CNIL) (no. 2229845 v.0), in compliance with French data protection legislation.

The study included consecutive adult patients with active pCD and active luminal CD treated with upadacitinib at the time of treatment initiation. Patients who did not receive the standard induction dose of 45 mg once daily, those with inactive disease, and those treated for postoperative recurrence prevention were excluded. Upadacitinib was prescribed in accordance with the recommendations of the Pharmacovigilance Risk Assessment Committee of the European Medicines Agency. Active pCD at baseline was defined at the time of upadacitinib initiation by the presence of clinically active perianal primary or secondary lesions, as determined by physical examination and classified according to the Cardiff classification [12]. Primary lesions were defined by at least one visible anal ulceration or fissure and secondary lesions by one or more actively draining perianal fistulas. Active luminal CD was defined by Harvey‐Bradshaw index (HBI) > 4 and/or at least one objective sign of active inflammation [C‐reactive protein (CRP) ≥ 5 mg/L, or faecal calprotectin ≥ 250 μg/g, or simple endoscopic score for CD (SES‐CD) ≥ 4, or bowel thickness > 3 mm in magnetic resonance imaging (MRI) or intestinal ultrasound (IUS)].

Upadacitinib was administered orally at an induction dose of 45 mg once daily for 12 weeks. After 12 weeks, the maintenance regimen (15 or 30 mg once daily) was selected at the discretion of the treating physician. During the maintenance phase, a reinduction course—consisting of upadacitinib 45 mg daily for a minimum of 3 months—could be given based on clinical judgement.

### 2.2 Data Collection

Data were collected using an electronic case report form completed by investigators at each participating centre based on a review of patients' medical charts. Baseline data included demographic characteristics [sex, age, body mass index (BMI), smoking status], CD medical and surgical history (including presence of a draining seton), prior drug exposure, CD location and phenotype according to the Montreal classification, clinical activity using Harvey Bradshaw Index (HBI) and Patient Reported Outcome 2 (PRO2) score, and the type of perianal lesions. According to the American gastroenterological association, a complex fistula was defined as one that is high and/or associated with multiple external openings, a perianal abscess, a rectovaginal fistula, an anorectal stricture or macroscopic evidence of rectal inflammation. In contrast, a simple fistula was defined as a low fistula with a single external opening, in the absence of features characteristic of a complex fistula [13].

All included patients were followed according to routine practice. Resolution of drainage and healing of anal ulcerations and/or fissures were assessed at week 12, 24 and 52. Routine laboratory parameters, including haemoglobin, and CRP were recorded at each visit. Information regarding upadacitinib treatment (dosage, discontinuation and reasons for discontinuation), concomitant use of corticosteroids, antibiotics, topical therapies, other advanced therapy or immunomodulators as well as the presence of a draining seton and the need for CD‐related surgery or hospitalization was documented at each visit. Perianal MRI performed during follow‐up, as well as all adverse events (AEs) were also recorded.

### 2.3 Study Outcomes

The primary observational outcome was clinical remission of pCD at week 52 defined as the absence of draining pus (including upon pressure) and healing of anal ulcerations and/or fissures, without the addition of new dedicated treatment for perianal lesions (antibiotics and/or topics), or initiation of new advanced therapy. Secondary outcomes included: clinical remission of pCD at week 12 and 24; clinical response at week 12, 24 and 52, defined as improvement of at least 50% of fistulas and/or ulcerations by physician's assessment; MRI‐based remission and response, as assessed by local radiologist's, focussing on the size of perianal abscess (if present), T2 hyperintensity and T1 enhancement after gadolinium injection; necessity for surgical intervention for pCD or CD‐related hospitalization during follow‐up; complete withdrawal of setons if present at inclusion; and safety (AEs, treatment cessation due to AEs). Serious AEs (SAEs) were defined as those resulting in treatment interruption, hospitalization, disability, persistent damage, digestive surgery or death. Patients with upadacitinib discontinuation, initiation of a new advanced therapy during follow‐up or need for additional perianal surgery were considered in failure for all clinical and radiological outcomes.

## 3 Statistics

Categorical variables were expressed as numbers (percentages), and continuous variables were summarized as medians with interquartile ranges (IQR). The 95% confidence intervals (CIs) for proportions were calculated using the Wilson‐Brown method. A non‐response imputation approach was applied for all clinical outcomes: patients lost to follow‐up or with missing data at the follow‐up visit were considered treatment failures. No imputation was applied to radiological outcomes, which were described as observed (AO). A sensitivity analysis using the AO approach was also performed for clinical outcomes. Categorical variables were compared using the chi‐square test. Univariate logistic regression analysis was conducted to identify predictors of clinical remission of fistulizing pCD at week 52, with results expressed as odds ratio (OR) and 95% CIs. Cut‐off values for continuous variables were chosen to maximize the likelihood ratio and were based on clinical relevance. All analyses were two‐tailed, and a *p* < 0.05 was considered statistically significant.

## 4 Results

### 4.1 Study Population

A total of 59 patients with active pCD were included: 43 with fistulizing pCD and 16 with isolated anal ulceration. Among patients with fistulizing pCD, 14/43 (33%) had draining seton at the time of upadacitinib initiation. Baseline characteristics are summarized in Table 1. Briefly, at baseline, median age was 38 years (IQR: 29–47), and 29 patients (49%) were male. The median duration since CD diagnosis was 13 years (8–19). More than two thirds of patients had undergone at least one previous perianal surgery, including 34/43 (79%) in the subgroup of fistulizing pCD. The most frequent luminal disease location was ileocolonic (54%), and the most frequent phenotype was non‐stricturing, non‐penetrating (45%). All patients had previously been exposed to at least one advanced therapy before upadacitinib initiation (median 4 [3–5]), with 95% having failed at least two biologics, and 88% having failed infliximab (Figure S1). Concomitant corticosteroid use at induction was noted in 15% of patients.

**TABLE 1:** Clinical characteristics of the 59 patients with perianal Crohn's disease at initiation of upadacitinib treatment.

Characteristic	*n* = 59
Age, years [median (IQR)]	38 [29–47]
Male gender, *n* (%)	29 (49)
BMI, kg/m^2^ [median (IQR)]	24 [21–27]
Active smoker, *n* (%)	11 (19)
Duration since CD diagnosis, years [median (IQR)]	13 [8–19]
Location, *n* (%)	
Ileal	12 (20)
Colonic	15 (25)
Ileocolonic	32 (54)
Upper gastrointestinal tract involvement	5 (9)
Disease behaviour, *n* (%)	
Inflammatory	25 (45)
Stricturing	11 (20)
Penetrating	20 (36)
History of perianal surgery, *n* (%)	40 (68)
Drainage	36 (61)
Injection of stem cells	3 (5)
Curettage	5 (9)
Other	2 (3)
Type of active perianal lesions	
Primary lesions (fissures or ulcerations)	16 (27)
Secondary lesions: simple fistula*	15 (25)
Secondary lesions: complex fistula*	28 (48)
Prior medications exposure, *n* (%)	
Thiopurine	45 (76)
Methotrexate	28 (48)
Infliximab	52 (88)
Adalimumab	50 (85)
Golimumab	14 (24)
Certolizumab	5 (9)
Ustekinumab	53 (90)
Vedolizumab	38 (64)
Risankizumab	5 (9)
Guselkumab	4 (7)
Number of prior biologics [median (IQR)]	4 [3–5]
Patients with stoma, *n* (%)	7 (12)
Patients with drainage seton, *n* (%)	14 (26)
Disease activity at baseline	
HBI [median (IQR)]	9 [6–13]
CRP, mg/L [median (IQR)]	21 [7–37]
Calprotectin, μg/g [median (IQR)]	1366.5 [477–2055]
Albumin, g/L [median (IQR)]	37 [33–42]
Concomitant medications, *n* (%)	
Steroids	9 (15)
Immunosuppressants	0
Other biologic	2 (3)

* According to American gastroenterological association classification.

Abbreviations: BMI, body mass index; CD, Crohn's disease; CRP, C‐reactive protein; HBI, Harvey Bradshaw Index.

### 4.2 Persistence of Upadacitinib Therapy

After a median follow‐up duration of 49 weeks (IQR, 46–52), 16/59 patients (27%) stopped upadacitinib. Among these patients, 14 (24%) stopped upadacitinib due to treatment failure, one (2%) for financial reasons and one for pregnancy. At week 52, upadacitinib treatment was maintained in 42/59 patients (71%).

**FIGURE 1:**
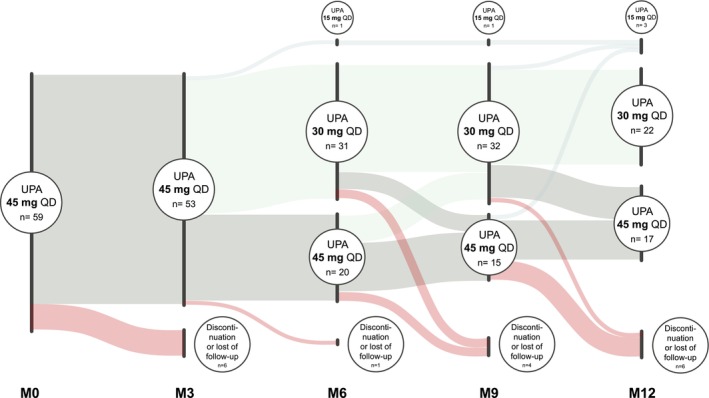
Sankey diagram illustrating upadacitinib dosing regimens over 1 year in patients with refractory perianal Crohn's disease in a multicentre cohort. M, months; OD, once a day.

After the 12‐week induction regimen with upadacitinib, 27/59 patients (46%) received a reinduction course of 45 mg once daily within the 1‐year study period, including 20/27 patients (74%) who maintained the induction dose beyond the initial 12‐week induction phase (Figure 1). During the 1‐year study period, the median duration of treatment with 45 mg once daily was 12 weeks (12–39). In patients receiving a conventional maintenance regimen, a daily dose of 30 mg was used in most patients (Figure 1).

### 4.3 Clinical Effectiveness of Upadacitinib Therapy

**FIGURE 2:**
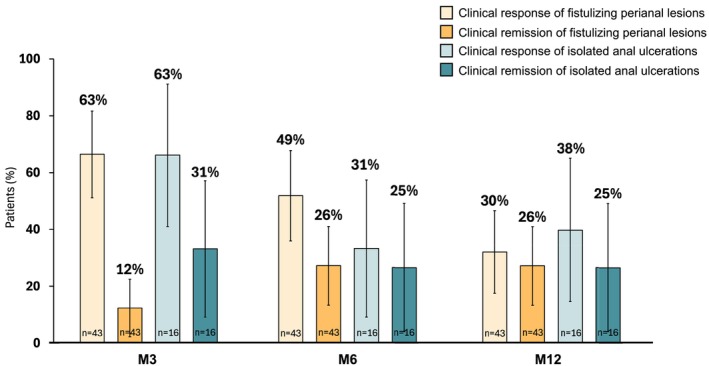
Clinical response to upadacitinib treatment in refractory perianal Crohn's disease in a multicentre cohort at 3, 6 and 12 months.

At week 52, clinical remission of pCD was achieved in 15/59 patients (25%) and clinical response in 19/59 patients (32%). In the subgroup of patients with fistulizing pCD, 11/43 patients (26%) achieved clinical remission, and 13/43 patients (30%) achieved clinical response (Figure 2). In the subgroup of patients with isolated anal ulcerations, clinical remission was observed in 4/16 patients (25%) and clinical response in 6/16 patients (38%) (Figure 2).

A sensitivity analysis excluding patients lost to follow‐up or with missing data showed clinical remission and clinical response rates of 11/33 (33%) and 13/33 (39%), respectively, among patients with fistulizing pCD. In patients with isolated anal ulcerations, clinical remission and response were achieved in 4/11 (25%) and 6/11 patients (38%), respectively (Figure S2).

Clinical outcomes were also evaluated at a median 12 weeks (9–15) and 26 weeks (24–30) after initiation of upadacitinib. Clinical remission was observed in 5/43 (12%) and in 11/43 (26%) patients with fistulizing pCD at 12 and 24 weeks, respectively, and clinical response in 27/43 (63%) weeks and in 21/43 (49%) after 24 weeks (Figure 2). Response and remission rates were similar in patients with isolated anal ulcerations (Figure 2). Rates of clinical remission and response at week 12 were comparable between patients who underwent at least one re‐induction regimen and those who did not receive re‐induction (19% versus 19% and 63% versus 74%, *p* = 1 and 0.5, respectively).

Among the 14 patients with draining seton at baseline, seton removal was possible in 4 (29%) patients by week 52. During this period, 10/59 patients (17%) underwent perianal surgery, and 11/59 patients (19%) had a pCD‐related hospitalization.

### 4.4 Factors Associated With Clinical Remission at Week 52

**FIGURE 3:**
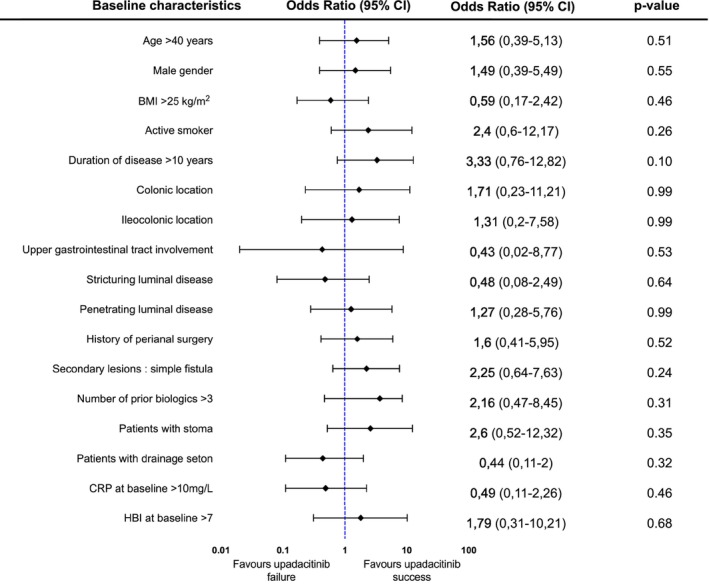
Forest plot of odds ratios for predictors of clinical remission in fistulizing perianal Crohn's disease at week 52. Simple of complex fistula was defined according to the American gastroenterological association classification.

In univariable analysis, no clinical parameter was associated with clinical remission of fistulizing pCD at week 52 (Figure 3). Notably, among the 27 patients who underwent at least one re‐induction regimen, the remission and response rate of pCD was 9/27 (33%) and 11/27 (41%) at week 52, respectively, which were not significantly different from those observed in patients receiving a conventional maintenance regimen of 15 or 30 mg daily (*p* = 0.29 and *p* = 0.30, respectively).

### 4.5 Radiological Outcomes

Radiological response was assessed in 13 patients with fistulizing pCD after a median of 29 weeks (22–49). Radiological response was observed in 9/13 (69%), and clinical remission in 1/13 (8%).

### 4.6 Safety

During follow‐up, a total of 17 AEs were reported in 15 patients (25%) (Table 2). The most frequent AE was acne, affecting seven patients (12%). Three patients (5%) experienced SAEs, all of which corresponded to CD exacerbation. No malignancies, deaths, thromboembolic events, major adverse cardiovascular events, severe infections or zoster cases were observed.

**TABLE 2:** Adverse events observed during the 52‐week upadacitinib treatment in 59 patients with perianal Crohn's disease.

Variables	Number of events (%)
Participants with ≥ 1 adverse event during the first 12 weeks	14 (24)
Adverse event	
Acne	7 (12)
Anal pain	1 (2)
Cytolysis	1 (2)
Alopecia	1 (2)
ENT infection	1 (2)
Dyslipidaemia	2 (3)
Serious adverse event	
CD exacerbation	3 (5)

Abbreviations: CD, Crohn's disease; ENT, nose and throat.

## 5 Discussion

This nationwide multicentre cohort of 59 patients is the first to evaluate the long‐term real‐world effectiveness of upadacitinib in pCD. At 1 year, clinical remission of fistulizing pCD was observed in 26% of patients, and clinical response in 30%, with similar outcomes for patients with isolated anal ulcerations.

While infliximab is the standard treatment for pCD, only one‐third of these patients achieve long‐term remission [6]. To date, several alternatives are available, including ustekinumab, anti‐IL23p19 agents and upadacitinib. In the absence of randomized controlled trials in this specific population, real‐world data can help guide therapeutic strategies. Concerning upadacitinib, only a post hoc analysis of the pivotal upadacitinib trials in luminal CD reports the efficacy of upadacitinib in pCD. Among the 1021 CD patients, 56 had draining fistula at baseline, respectively, 18%, 16% and 0% of patients receiving upadacitinib 15, 30 or placebo achieved closure of external openings, and 28%, 23% and 0% resolution of drainage of perianal fistulas in maintenance, respectively [9]. Notably, our real‐world population includes a cohort of more refractory patients, all having failed at least one prior advanced therapy and having a longer median disease duration. In this context, our findings confirm that UPA is positioned as a therapeutic alternative in pCD when anti‐TNF treatment has failed. Remission rates were comparable to—or higher than—those reported for other advanced therapy in pCD failing anti‐TNF. For instance, in real‐world cohorts of highly refractory pCD patients, the week 52 remission rate was 33% with risankizumab [14]; 23% at week 24 with vedolizumab [15], and 39% at week 48 with ustekinumab [16]. It is important to note that none of these studies employed NRI analysis, and therefore, these rates should be compared with the 33% clinical remission reported in our AO analysis.

In our cohort, a high proportion of patient (46%) received at least one reinduction regimen (45 mg daily) after induction. In most cases, the reinduction was administered immediately following induction without any dose tapering, suggesting that an insufficient clinical response as judged by the treating physician was the main reason for reinduction. We observed no additional clinical benefit or safety concerns with the reinduction strategy compared with the conventional maintenance regimen (primarily 30 mg daily). These findings are consistent with phase 3 post hoc analyses, which showed no clear dose–efficacy relationship for pCD patients [9]. Few data are available on the efficacy of the reinduction strategy in real‐world contexts. Nevertheless, one prospective cohort of inflammatory bowel disease (IBD) patients has already reported an encouraging efficacy (up to 80%) and safety profile in a different context: recapturing response in luminal CD following clinical relapse after a switch to a lower maintenance dose [17].

During follow‐up, a substantial proportion of patients lost their response with a response rate declining from 63% at 3 months to 49% and 30% at 6 and 12 months, respectively. A similar trend was observed with upadacitinib in a recent retrospective study, in which upadacitinib was associated with a significantly greater hazard of treatment discontinuation through 52 weeks than risankizumab [18]. Likewise, a Spanish retrospective cohort study of more than 500 patients reported lower persistence with upadacitinib than with risankizumab [19]. These real‐world data are consistent with findings from the phase 3 maintenance trial U‐ENDURE, in which patients with Crohn's disease who achieved a clinical response at week 12 maintained response at week 52 in 51% and 41% of cases with 30 mg and 15 mg daily maintenance regimens, respectively [8]. Taken together, these findings suggest a dose‐dependence effect, as well as the possible emergence of alternative inflammatory pathways during the maintenance phase.

The safety assessment was limited in this cohort by the sample size and the absence of follow‐up after 1 year. In our experience, upadacitinib was safe for the treatment of pCD. The most frequent AE was acne, reported in 12% of patients. A similar incidence of acne has been reported in previous studies [8, 11, 20, 21]. In contrast, a higher incidence of acne (43%) was observed in IBD patients undergoing upadacitinib reinduction in an observational study [17]. No death, thromboembolic events, major adverse cardiovascular events, severe infections, or zoster cases were reported.

This nationwide cohort is the first one reporting the 1‐year effectiveness of upadacitinib as therapy for pCD in a real‐world setting. The sample size, limited to 59 patients, is comparable to that included in the post hoc analysis of the phase 3 trial [9]. Strengths of this study include the 1‐year follow‐up and the multicentre design. However, we acknowledge that the retrospective design is the main limitation. In order to mitigate this bias, all consecutive patients in all participating centres were included. Moreover, we chose to perform NRI analysis to minimize the non‐response bias. Furthermore, the clinical endpoints chosen, particularly the presence or absence of draining pus, were subject to the physician's assessment, limiting reproducibility and objectivity—an inherent challenge in all clinical studies on pCD. A recent expert consensus has therefore recommended focusing treatment goals on quality‐of‐life endpoints [22], which themselves may be subject to criticism [23]. To address this limitation partially, MRI data were collected in a subset of patients, though not centrally read, to objectively confirm the clinical efficacy signal. Finally, the sample size was insufficient to identify any predictors of treatment response.

In conclusion, the observed 1‐year clinical remission rate of 26% in fistulizing pCD supports the potential of upadacitinib as a treatment option in anti‐TNF refractory pCD patients. Comparative studies between upadacitinib, ustekinumab and risankizumab are warranted to establish the optimal therapeutic strategy in this population.

## Author Contributions

**Nicolas Richard:** writing – original draft, data curation, software, formal analysis. **David Laharie:** writing – review and editing, data curation, project administration, resources. **Benoît Coffin:** writing – review and editing, data curation. **Anne‐Laure Pelletier:** data curation, writing – review and editing. **Mathurin Fumery:** writing – review and editing, writing – original draft, methodology, data curation, supervision, resources. **Maria Nachury:** data curation, writing – review and editing. **Morgane Amil:** writing – review and editing, data curation. **Aurélien Amiot:** writing – review and editing, data curation. **Romain Altwegg:** writing – review and editing, data curation, conceptualization. **Anne Bozon:** writing – original draft, writing – review and editing, conceptualization, methodology, data curation, validation.

## Funding

The authors have nothing to report.

## Conflicts of Interest

N.R. has received lecture/consultant fees from AbbVie, Amgen, Celltrion, Ferring, Janssen, Lilly, Sandoz and Takeda. P.S. received consulting fees from Takeda, Abbvie, Merck‐M.S.D., Biocodex, Janssen, Amgen, Celltrion and Pfizer and grants from Biocodex and Janssen. R.A. has received lecture/consultant fees from AbbVie, Ferring, M.S.D., Celltrion, Biogen, Amgen, Sandoz, Pfizer, Alfasigma, Janssen and Takeda. M.N. received board membership, consultancy or lecture fees from Abbvie, Amgen, Biogen, Celltrion, Ferring, Fresenius‐Kabi, Galapagos, Janssen, Lilly, Mayoli‐Spindler, M.S.D., Nordic Pharma, Pfizer, Takeda and Viatris. D.L. declares counselling, boards, transports or fees from Abbvie, Amgen, Biocon, Celltrion, Ferring, Galapagos, Janssen, Lilly, Medac, M.S.D., Pfizer, Sandoz, Takeda. S.N. has received lecture/consultant fees from AbbVie, Ferring, Tillotts, Amgen, Fresenius, Sandoz, Pfizer, Celltrion, Gilead, Galapagos, Janssen, Medac and Takeda. B.C. has received consulting fees from Lilly. A.‐L.P. has received lecture/consultant fees from Janssen, Pfizer and Novartis. M.U. declares counselling, boards or fees for AbbVie, Amgen, Celltrion, Janssen, Lilly, Takeda. A.A. received consulting fees from Abbvie, Lilly, M.S.D., Pfizer, Takeda, Tillotts Pharma, Adacyte, Janssen and Sandoz as well as lecture fees and travel accommodations from Abbvie, Lilly, Janssen, Pfizer, Takeda, Biogen, Fresenius Kabi, Adacyte, Amgen and Celltrion. L.V. has received fees for lectures and/or consulting fees from Abbvie, Amgen, Johnson & Johnson, Celltrion, Takeda, Pfizer, Lilly, Ferring, M.S.D., Dr. Falk Pharma, Nordic Pharma, Alpha sigma. M.F. has received lecture/consultant fees from AbbVie, Ferring, Tillotts, M.S.D., Biogen, Amgen, Fresenius, Hospira, Sandoz, Pfizer, Celgene, Gilead, Boehringer, Galapagos, Janssen and Takeda. A.B. has received consultant fees from AbbVie, Amgen, Pfizer, Janssen and Takeda. M.A. state that they have no competing interests regarding this work to disclose.

## Supporting information


**Figure S1:** UpSet plot demonstrating advanced therapy exposure prior upadacitinib initiation. ADA, adalimumab; CERT, certolizumab; GOL, golimumab; GUS, guselkumab; IFX, infliximab; RIS, risankizumab; UST, ustekinumab; VEDO, vedolizumab.

**Figure S2:** Clinical response to upadacitinib treatment in refractory perianal Crohn's disease in a multicentre cohort at 3, 6 and 12 months as observed analysis.

## Data Availability

The data that support the findings of this study are available on request from the corresponding author. The data are not publicly available due to privacy or ethical restrictions.
