# Supplementary figures and images for: One‐Year Effectiveness of Upadacitinib in Perianal Crohn's Disease: A Real‐World GETAID Study
Source: Aliment Pharmacol Ther. 2026 Apr 27;64(2):235–42. doi: 10.1111/apt.70682 (PMC13309209; doi:10.1111/apt.70682)

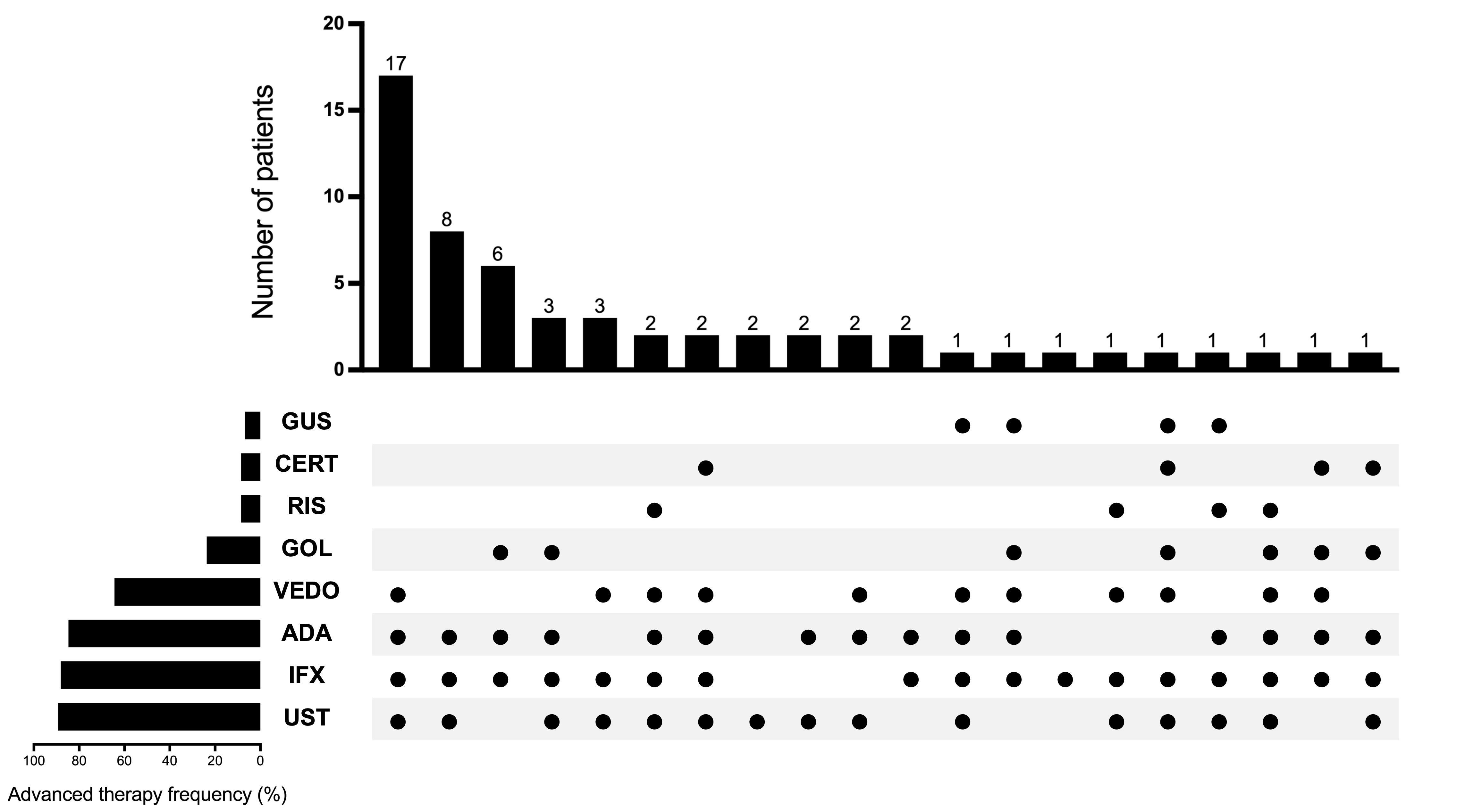

Supplement: Supplementary file 1 — Figure S1: UpSet plot demonstrating advanced therapy exposure prior upadacitinib initiation. ADA, adalimumab; CERT, certolizumab; GOL, golimumab; GUS, guselkumab; IFX, infliximab; RIS, risankizumab; UST, ustekinumab; VEDO, vedolizumab. [file APT-64-235-s001.jpg]

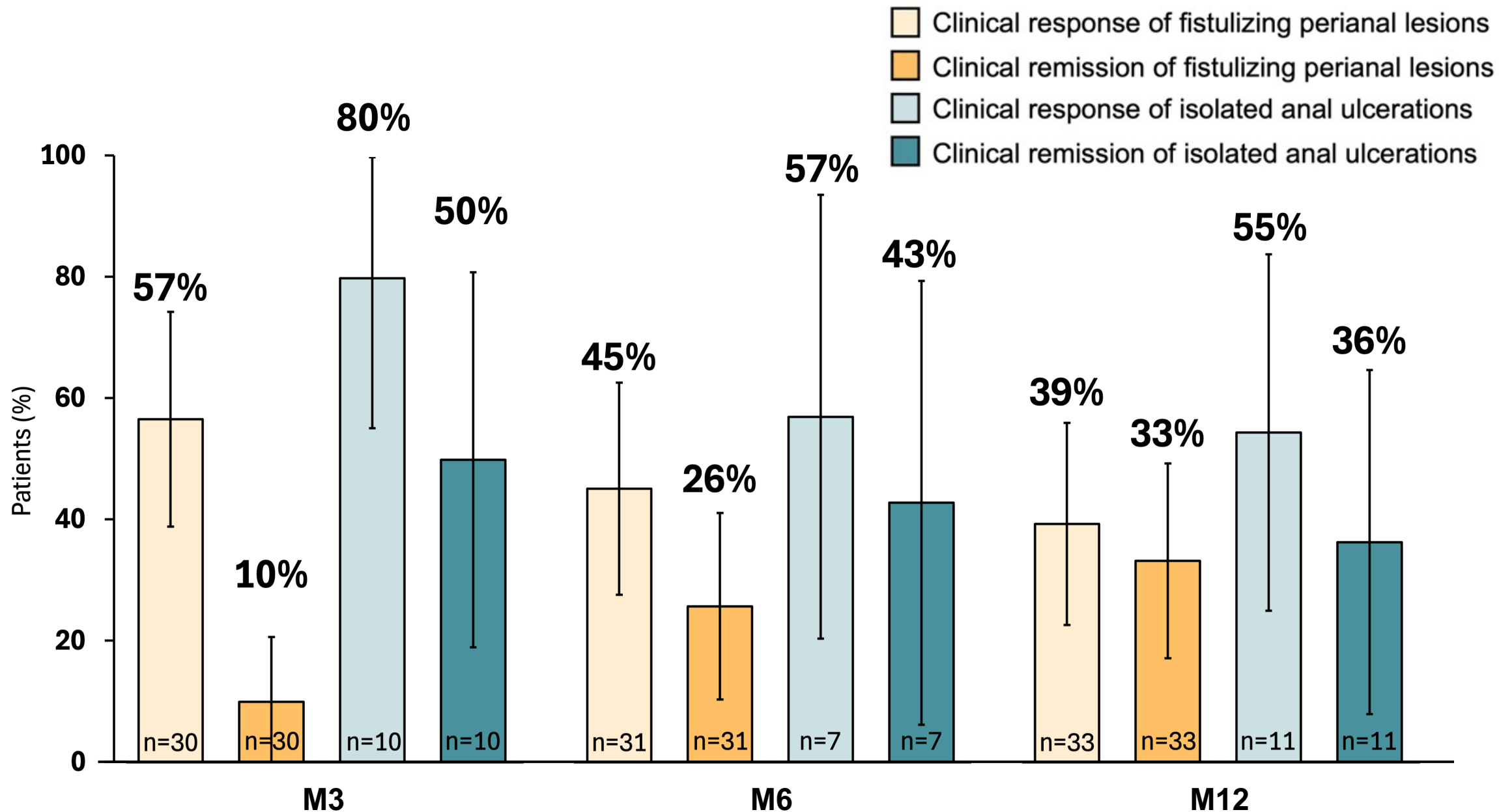

Supplement: Supplementary file 2 — Figure S2: Clinical response to upadacitinib treatment in refractory perianal Crohn's disease in a multicentre cohort at 3, 6 and 12 months as observed analysis. [file APT-64-235-s002.pdf]
